# Association of Electronic Health Record Inbasket Message Characteristics With Physician Burnout

**DOI:** 10.1001/jamanetworkopen.2022.44363

**Published:** 2022-11-30

**Authors:** Sally L. Baxter, Bharanidharan Radha Saseendrakumar, Michael Cheung, Thomas J. Savides, Christopher A. Longhurst, Christine A. Sinsky, Marlene Millen, Ming Tai-Seale

**Affiliations:** 1 Division of Ophthalmology Informatics and Data Science, Viterbi Family Department of Ophthalmology and Shiley Eye Institute, University of California, San Diego, La Jolla; 2 Department of Medicine, University of California, San Diego, La Jolla; 3 Department of Family Medicine, University of California, San Diego, La Jolla; 4 Department of Pediatrics, University of California, San Diego, La Jolla; 5 American Medical Association, Chicago, Illinois

## Abstract

**Importance:**

Physician burnout is an ongoing epidemic; electronic health record (EHR) use has been associated with burnout, and the burden of EHR inbasket messages has grown in the context of the COVID-19 pandemic. Understanding how EHR inbasket messages are associated with physician burnout may uncover new insights for intervention strategies.

**Objective:**

To evaluate associations between EHR inbasket message characteristics and physician burnout.

**Design, Setting, and Participants:**

Cross-sectional study in a single academic medical center involving physicians from multiple specialties. Data collection took place April to September 2020, and data were analyzed September to December 2020.

**Exposures:**

Physicians responded to a survey including the validated Mini-Z 5-point burnout scale.

**Main Outcomes and Measures:**

Physician burnout according to the self-reported burnout scale. A sentiment analysis model was used to calculate sentiment scores for EHR inbasket messages extracted for participating physicians. Multivariable modeling was used to model risk of physician burnout using factors such as message characteristics, physician demographics, and clinical practice characteristics.

**Results:**

Of 609 physicians who responded to the survey, 297 (48.8%) were women, 343 (56.3%) were White, 391 (64.2%) practiced in outpatient settings, and 428 (70.28%) had been in medical practice for 15 years or less. Half (307 [50.4%]) reported burnout (score of 3 or higher). A total of 1 453 245 inbasket messages were extracted, of which 630 828 (43.4%) were patient messages. Among negative messages, common words included medical conditions, expletives and/or profanity, and words related to violence. There were no significant associations between message characteristics (including sentiment scores) and burnout. Odds of burnout were significantly higher among Hispanic/Latino physicians (odds ratio [OR], 3.44; 95% CI, 1.18-10.61; *P* = .03) and women (OR, 1.60; 95% CI, 1.13-2.27; *P* = .01), and significantly lower among physicians in clinical practice for more than 15 years (OR, 0.46; 95% CI, 0.30-0.68; *P* < .001).

**Conclusions and Relevance:**

In this cross-sectional study, message characteristics were not associated with physician burnout, but the presence of expletives and violent words represents an opportunity for improving patient engagement, EHR portal design, or filters. Natural language processing represents a novel approach to understanding potential associations between EHR inbasket messages and physician burnout and may also help inform quality improvement initiatives aimed at improving patient experience.

## Key Points

**Question:**

Are electronic health record (EHR) inbasket message characteristics, such as volume (number of messages), length (word count), and sentiment (positive, neutral, or negative), associated with physician burnout?

**Findings:**

In this cross-sectional study using natural language processing (NLP) to analyze 1 453 245 messages received by 609 physicians, there were no significant associations between message characteristics and burnout. Analysis of negative messages revealed frequent use of expletives and words related to violence.

**Meaning:**

These findings suggest that message characteristics may not be associated with physician burnout, but NLP can facilitate identification of negative messages and inform subsequent interventions, such as automated inbasket filters, root cause analysis of patient frustrations, and efforts to improve patient experience.

## Teaser

This cross-sectional study evaluates the association between electronic health record inbasket message characteristics and physician burnout using a physician survey and natural language processing analysis of messages.

## Introduction

Burnout among physicians and other health care professionals has been increasingly well-recognized.^1,2,3,4,5,6^ Burnout concerns continue to be pronounced with the COVID-19 pandemic.^7,8,9,10^ Physician burnout has been associated with widespread adoption of electronic health records (EHRs) according to studies^11,12,13,14,15^ that have demonstrated the time, cognitive burden, and stress associated with using EHRs and other health information technology (IT) systems. Of particular interest is understanding the burden of the EHR “inbasket,” which serves as an integrated messaging platform and is an available functionality for several EHR vendor systems. Message types can include laboratory or imaging results, messages from other physicians or staff members, patient messages, and system-generated messages (eg, incomplete progress notes or system downtimes). Prior studies^16,17,18^ have shown that high message volumes and time incurred in inbasket management have been associated with physician burnout.

The inbasket’s role is growing with increasing adoption of EHR patient portals and data sharing with patients via federal mandates such as the 21st Century Cures Act.^19^ These portals have facilitated direct messaging between patients and physicians, and the widespread availability of health data has generated increasing messaging volumes.^20^ Furthermore, increased adoption of telemedicine in response to the COVID-19 pandemic also increased use of inbasket messages for communication^21,22^ A recent analysis^23^ showed that EHR inbasket messages increased by 157% during the pandemic.

The association between message sentiment and burnout has not been well studied. Prior evidence in other fields has suggested that individuals are less inhibited during electronic/online communication compared with in-person communication and more likely to engage in aggressive or negative language, even when not anonymous.^24,25,26^ Subsequently, in this study we extracted EHR inbasket messages and used a natural language processing (NLP) approach to evaluate whether inbasket message sentiment may be associated with physician burnout.

## Methods

### Study Population

The study population consisted of attending physicians at University of California San Diego Health who participated in an online survey of well-being and EHR use between April and September 2020 and had inbasket messages in our institutional EHR system (Epic, EpicCare Systems). All participants provided written informed consent. The study was approved by the University of California San Diego institutional review board, adhered to the Declaration of Helsinki,^27^ and followed the Strengthening the Reporting of Observational Studies in Epidemiology (STROBE) reporting guideline.^28^

### Physician Burnout Survey

An invitation to a confidential online survey (Qualtrics) was sent to all eligible physicians in April 2020. Reminders were sent in May, July, and August 2020 before closure in September 2020. Participants were surveyed regarding characteristics such as self-reported gender, race, ethnicity, work setting (inpatient or outpatient), specialty, clinical hours (clinical full-time equivalent [FTE] ≥50% vs <50%), and duration of experience (≤15 years or >15 years). Gender, race, and ethnicity information was self-reported according to investigator-defined categories (gender: Female, Male, Other, or prefer not to answer; race: Asian, Black or African American, Other or prefer not to answer, or White; ethnicity: Hispanic or Latino, Not Hispanic or Latino, or Prefer not to answer). There was no secondary categorization of the “Other or prefer not to answer” category by investigators; this was self-reported by participants if they identified with a group not listed in the prespecified options. These factors were included in the study to assess whether they may be associated with burnout and to serve as a covariate for the evaluation of message sentiment.

To assess overall burnout, the survey included the mini-Z single-item burnout measure, a standardized instrument for measuring burnout,^29^ which asks participants to self-report their level of burnout on a scale of 1 to 5 and has been widely used previously in physician burnout research.^17,30,31^ This single-item burnout measure correlates mainly with the emotional exhaustion domain of burnout.^32^ The full survey instrument is available in the eAppendix in Supplement 1. The burnout item in the original survey instrument was coded with 5 representing no symptoms of burnout (listed first) and 1 representing completely burned out (listed last), consistent with the format cited in a prior study by Linzer et al.^33^ However, because the online survey vendor automatically coded the first option for any categorical variable as 1, the output from the survey was transformed to the following scale: 1, “I enjoy my work. I have no symptoms of burnout”; 3, “I am beginning to burn out and have one or more symptoms of burnout, e.g. emotional exhaustion”; and 5, “I feel completely burned out. I am at the point where I may need to seek help.” We used this transformed survey output and scale for all subsequent analyses. With this scale, burnout is defined as a score of 3 or higher, which is what we used as our threshold for the primary outcome.

### Extraction and Processing of EHR Inbasket Data

The survey was confidential, but not anonymous, to allow linkage with inbasket messages. We queried the EHR clinical data warehouse to extract messages sent to survey respondents during the yearlong period preceding their survey completion date. We also extracted information regarding message type according to existing classifications in the EHR system. Our analyses included those of all messages as well as those focused on patient messages specifically. We defined patient messages as messages with the following vendor-provided classifications: Patient Medical Advice Request, MyChart Notifications, and Patient Calls. Word counts were derived for each message.

### NLP for Sentiment Analysis

In preparation for sentiment analysis, message headers, stop words, and numbers were removed, and words were lemmatized using the Python package *nltk*, a natural language toolkit.^34^ Lemmatization involves grouping inflected forms of a word together as the base word or lemma (eg, *meet* and *meeting* can be lemmatized to *meet*). The processed messages were fed through VADER, a rule-based sentiment analysis model that generates a score for each message ranging from –1.0 (extremely negative) to 1.0 (extremely positive).^35^ We used the distribution of sentiment scores to determine thresholds for positive and negative boundaries (eFigure in Supplement 1); a sentiment score less than -0.1 was defined as negative, and sentiment scores more than 0.1 were positive. The remaining messages were defined as neutral (ie, from −0.1 to 0.1). Using these definitions, each message was classified as positive, negative, or neutral. We manually reviewed a subset of 100 messages in each category to verify sentiment analysis classification. Examples of positive and negative messages are provided in the eTable in Supplement 1. Because our objective was to evaluate message sentiment at scale and investigate potential associations of overall negativity in messages with burnout, we included all negative messages according to the computationally derived sentiment score and not only those with negative content specifically directed at physicians.

### Visualizations of Words Among InBasket Messages

We generated bar graphs depicting the top 100 most common words and their frequencies among positive and among negative patient messages using the WordCloud Python package.^36^ Expletives were censored by replacing letters with asterisks. Of note, the sentiment score is not based solely on word content. Several additional factors are incorporated, including use of negation, punctuation (eg, exclamation points to indicate intensity), word shape (eg, ALL CAPS to indicate stronger emotion), degree modifiers (eg, *very* or *kind of*), use of emoticons/emojis, and acronyms. Although words alone may not account for the sentiment scores, we undertook these visualizations to facilitate interpretability.

### Statistical Analysis

Univariate descriptive analyses were conducted to evaluate the characteristics of participating physicians and the distribution of burnout ratings. Extracted inbasket messages were grouped by physician. Aggregated variables such as mean sentiment score; mean proportions of positive, negative, and neutral messages; total number of messages; and mean word counts were created. Bivariate unadjusted analyses investigated the associations between message sentiment scores and burnout using analysis of variance testing. Finally, we performed multivariable logistic regression modeling adjusting for physician demographics and other characteristics using the glm (generalized linear models) package in R, using the binomial family argument in glm to represent the binary burnout outcome (burnout is indicated by scores of 3-5, and no burnout is indicated by scores of 1-2). Modeling was performed for all inbasket messages, as well as for the subset of patient messages only. NLP was performed in Python version 3.7.1, while statistical analyses were conducted using R statistical software version 4.0.3 (R Project for Statistical Computing). Statistical significance was defined as 2-sided *P* < .05. Data were analyzed September to December 2020.

## Results

### Participating Physicians and Burnout Distribution

Of 1038 eligible attending physicians, 627 (60.4%) completed the survey. Of these, 609 had a total of 1 453 245 inbasket messages available for analysis (Table 1). Approximately half (297 [48.8%]) were women, slightly higher than administrative data regarding all eligible attending physicians at our institution (41.6% women). More than half (343 [56.3%]) of participants identified as White (Table 1), which was lower than the eligible population (67.1%). Almost two-thirds of participants (391 [64.2%]) practiced in outpatient settings, and the majority (428 [70.3%]) had practiced less than 15 years. A wide range of specialties were represented (Table 1). Half (307 [50.4%]) endorsed burnout with a mini-Z score of 3 or more (Table 1).

**Table 1.** Characteristics of Physician Respondents to a Burnout Survey at the University of California San Diego, Spring 2020

Characteristic	Respondents, No. (%) (N=609)
Burnout rating (Mini-Z)	
1 = I enjoy my work. I have no symptoms of burnout	98 (16.09)
2	204 (33.50)
3 = I am beginning to burn out and have one or more symptoms of burnout, eg, emotional exhaustion	253 (41.54)
4	49 (8.05)
5 = I feel completely burned out. I am at the point where I may need to seek help	5 (0.82)
Missing	0
Gender	
Female	297 (48.77)
Male	295 (48.44)
Other or prefer not to answer^a^	17 (2.79)
Missing	0
Race	
Asian	141 (23.15)
Black or African American	8 (1.31)
White	343 (56.32)
Other or prefer not to answer	117 (19.21)
Missing	0
Ethnicity	
Hispanic or Latino	37 (6.07)
Not Hispanic or Latino	505 (82.92)
Prefer not to answer	60 (9.85)
Missing	7 (1.15)
Work setting	
Outpatient	391 (64.20)
Inpatient	210 (34.48)
Missing	8 (1.31)
Clinical full-time equivalent	
≥50%	471 (77.34)
<50%	121 (19.87)
Missing	17 (2.79)
Years in medical practice	
≤15	428 (70.28)
>15	178 (29.23)
Missing	3 (0.49)
Specialty	
Family medicine	50 (8.21)
Surgery	50 (8.21)
Anesthesiology	48 (7.88)
Psychiatry	46 (7.55)
Emergency medicine	39 (6.40)
Radiology	39 (6.40)
Obstetrics and gynecology	39 (6.40)
General internal medicine	38 (6.24)
Hospital medicine	34 (5.58)
Hematology/oncology	32 (5.25)
Cardiovascular medicine	29 (4.76)
Other-fewer than 7 respondents^b^	26 (4.27)
Gastroenterology/hepatology	19 (3.12)
Pathology	17 (2.79)
Neurosciences	17 (2.79)
Pediatrics	16 (2.63)
Ophthalmology	14 (2.30)
Pulmonary, critical care, and sleep medicine	14 (2.30)
Dermatology	12 (1.97)
Infectious disease	11 (1.81)
Radiation medicine	11 (1.81)
Orthopedic surgery	8 (1.31)
Missing	0

^a^ There was no secondary categorization of the “Other or prefer not to answer” category by investigators; this was self-reported by participants if they identified with a group not listed in the prespecified options.

^b^ Specialties with fewer than 7 respondents were aggregated to prevent risk of re-identification.

### InBasket Message Characteristics

Examination of Epic Signal, a platform that provides off-the-shelf metrics regarding EHR use, demonstrated that the time in inbasket and volume of inbasket messages received at our institution is in the middle 50% of all institutions using Epic and not an outlier. When grouping messages by physician, the mean (SD) sentiment score was positive at 0.12 (0.05). The mean (SD) number of messages received per physician was 2389.9 (3169.4) in the preceding year. Of the total inbasket messages, 630 828 (43.4%) were patient messages. Among patient messages, the mean sentiment rating was also positive, with the mean (SD) sentiment score at 0.15 (0.15).

The most common words among positive messages (Figure, A) included words conveying gratitude or positive emotions, such as “thank,” “help,” “well,” “good,” “hope,” and “care.” Several abbreviations or informal/casual expressions were also among the common words, such as “fyi,” “ty,” “lol,” and “haha.”

**Figure.**
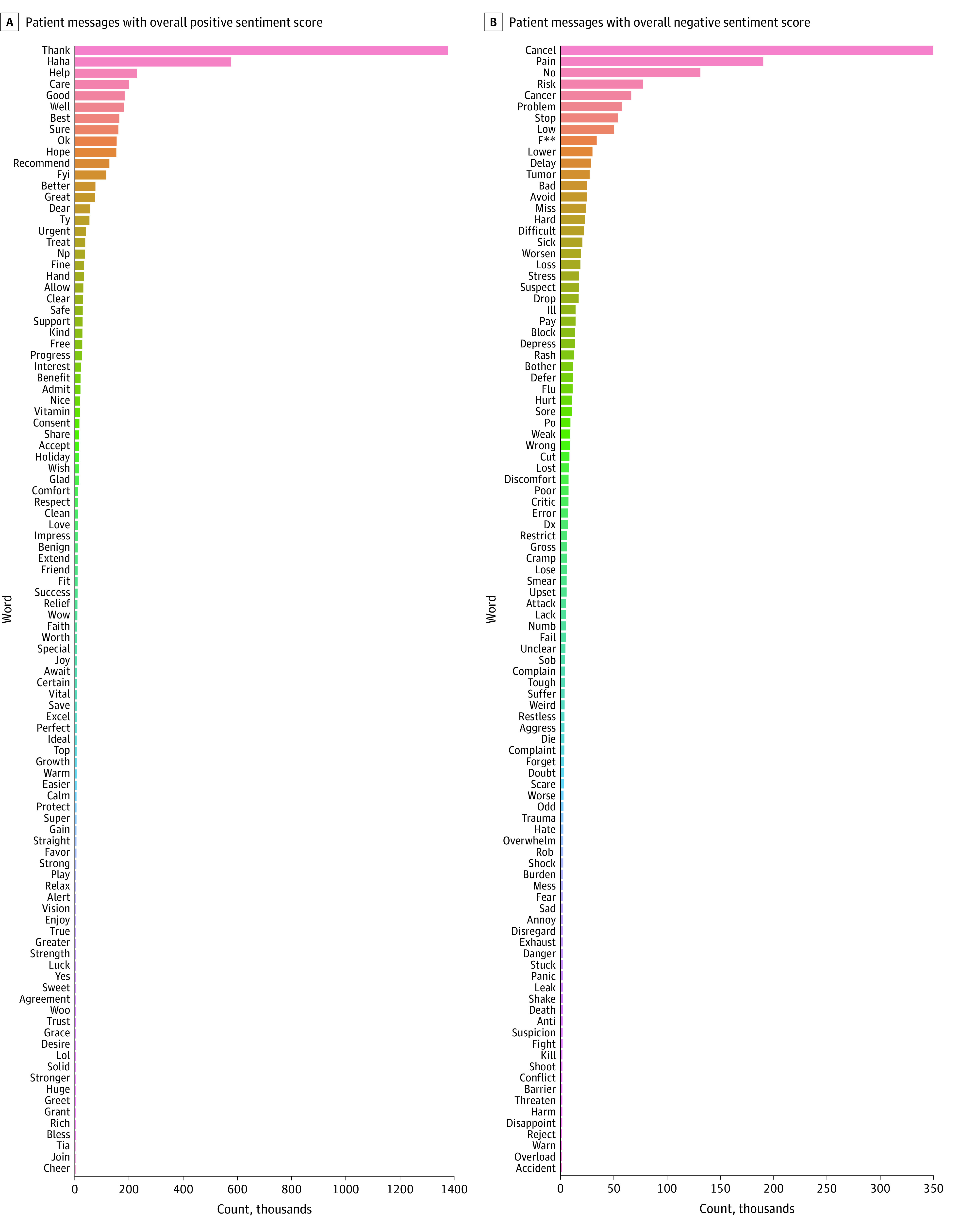
Bar Graphs Depicting the Most Common Words in Patient Messages With Overall Positive Sentiment Score and With Overall Negative Sentiment Score

Among negative messages, the most common words included “cancel,” “pain,” “no,” “cancer,” “problem,” and “stop” (Figure, B). Words describing medical/clinical entities were also fairly common. Of note, expletives were among the high-frequency words in negative messages, with f*** landing in the top 10 words overall (Figure, B). Table 2 includes examples of patient messages containing expletives.

**Table 2.** Examples of Electronic Health Record Inbasket Messages Sent From Patients Containing Expletives or Profanity

Word	Representative quotation
Cra*	“Got ultrasound today that shows a bunch of big nodes. The report is cra* and only describes one. Tall ipsilateral thyroid nodule not clearly malignant. I asked for an addended report.”
	“What’s happening with me you ask? In my latest news, I feel like cra* pretty much every day of my life and struggle completing basic human functions. So that’s fun for me.”
	“So I have to tell you I'm pretty perturbed by this whole thing. I don't care what the rules are, I think it's pretty cra**y, that there couldn't have been an exception regarding having the Covid test the morning before the procedure, considering all this cra* that could have been avoided, by you giving me the exact info, and your staff taking care of the insurance deal. Two trips up there again is a bit much. Why don't you see what you can do about it? If not, why don't you have one of these upper ups that make these rules give me a call.”
	“One more thing so you understand me. 23 y of severe chronic pain by itself has completely and totally kicked my ass!! It is so much to deal with by itself and a complete miracle I haven't committed suicide years ago. The feeling of being kicked in the balls 300 times a day and the feeling my nuts are in a vice 24/7 is just ridiculous and you have seen me take on so much other severely painful conditions that the combination is unbelievable!! Now I never stop with severe heavy wheezing and struggle to breathe constantly. I can't sleep and can't eat anything that doesn't get stuck in my throat. To me being terminally ill doesn't matter. To me it's can I do this cra* for ONE MORE DAY!! When I try to answer myself it is always No freaking way...”
He**	“As for how to communicate, playing email ping-pong via MyChart takes a he** of a lot longer and involves a lot more effort than a simple phone call. I always prefer to talk by phone.”
	“Why in the he** can I not get a report on my test Ct I had on Tuesday?????”
	“I would ask that you go ahead and prescribe the Lasix as I will be raising he** about the poor communication here.”
	“I need to find out as soon as possible the cost of the new drug that Dr *** is trying to help me get because it is very expensive but when I get sick for weeks at a time I will pay if this will make me well until we can get it at a discounted rate. I am on vacation and I have now been sick for three weeks so this is he**.”
	“Well I am not a religious person, I hope and expect that you will spend eternity in he**. You are an abusive, nasty, cheap person.”
	“This is a big GO TO HE** to ‘Doctor’ ***, whose cutting me off by refusing to fill my prescription for bupropuion on spite of multiple entreaties and his further silent abandonment of me caused me to force-wean myself from this medication. His lack of empathy and general lack of caring is disgusting and a violation of the spirit of the Hippocratic Oath he ostensibly took at some point. He definitely did harm here to me, and while this harm was not fatal or gave me any lasting injury, it was major discomfort. I plan to file a complaint against him with any medical boards and federal regulators I can find. I am sure that that won't do a damn thing, as patients have no recourse. I plan to also file complaints against his superiors at UCSD for allowing him and probably multiple other physicians under their control to be saddled with upwards of 200% or more of their patient capacities. Again, you all can go to he**.”
Bullsh**	“You and your university should be ashamed of yourselves. As a doctor, you should care about helping people. Here I sit 6 mos later, unable to pay my bills for rediculous blood tests that you ordered. UCSD is full of liars, hypocrites and I will do everything in my power to prevent anyone from going to your bullsh** office again. Thank you for $1600 in bills for 10-min of your time. I am ashamed that I wasted my time and money to visit you and UCSD. Just remember your ripping people off, not helping them get better. Congrats.”
	“And for you to hang up on me like you did today is totally unacceptable. I wasn't calling you names or threatening you. I was using adult language because I'm a fu**ing adult! And I had every right to be pissed having stayed up all night and morning only to find out you can't figure out how to use a fu**ing computer program that every one else at UCSD has no problem with. Even if ur messages are screened...they aren't deleted. All the doctors I've seen at student health can readily View MyChart messages. So I'm not buying that line of bullsh**!”
	“And don't give me some bullsh** about you needing to monitor me? Wtf does that mean? How have you been monitoring me since I've been seeing you?”
Fu**	“I am so upset that i was told the blood work would include the gender of the baby. I have been waiting 5 d to find it, and it wasnt even fu**ing tested!!!! What a disappointment in your office and the bullsh** i was told. I will be switching plans because this is sh**!”
	“This whole experience makes me want to just say fu** my health. I don't care what's wrong or what happens to me.”
	“What the actual fu**! What kind of PCP is this!”
	“Are you just put out with what's going on? This is serious too me and I am very concerned about. If this is how you're feeling about my issue that fu** it. You're not the one that is experiencing it!!!”

Abbreviations: UCSD, University of California San Diego; PCP, primary care physician.

### Message Characteristics and Burnout

Comparing all messages received by physicians reporting burnout (scores of ≥3 on the burnout question; 767 855 messages sent to 307 physicians) with those reporting no burnout (scores of 1-2; 685 390 messages sent to 302 physicians), those with burnout received a larger volume of messages (2501.2 vs 2269.5 messages per physician), although this did not reach significance (difference, 231.7 messages; 95% CI, 735.2-271.9 messages; *P* = .37) (Table 3). There were no significant differences in the message sentiment score, proportion of positive messages, proportion of negative messages, or mean word count (Table 3). Approximately one-half of messages were positive in both groups, and only approximately 5% of messages received were classified as negative in both groups, with the remainder being neutral.

**Table 3.** Comparison of Inbasket Message Volume and Sentiment According to Level of Physician Burnout

Message Characteristics	Messages among physicians, mean (SD)		95% CI for difference in means	*P* value
	Reporting burnout (score of 3 to 5)	Reporting no burnout (score of 1 or 2)		
All types of inbasket messages	767 855 messages sent to 307 physicians	685 390 messages sent to 302 physicians	NA	NA
Messages per physician, No.	2501.2 (3261.1)	2269.5 (3060.9)	−735.16 to 271.86	.37
Sentiment score	0.12 (0.05)	0.12 (0.05)	−0.01 to 0.01	.44
Proportion positive messages	0.50 (0.18)	0.47 (0.19)	−0.06 to 0.01	.10
Proportion negative messages	0.05 (0.06)	0.05 (0.05)	−0.01 to 0.01	.36
Word count	115.71 (42.03)	113.67 (36.21)	−8.29 to 4.19	.52
Patient messages only	338 242 messages sent to 275 physicians	292 586 messages sent to 256 physicians	NA	NA
Messages per physician, No.	1229.97 (1657.61)	1142.91 (1582.44)	−363.75 to 189.64	.54
Sentiment score	0.17 (0.07)	0.16 (0.05)	−0.02 to 0.002	.11
Proportion positive	0.69 (0.19)	0.67 (0.17)	−0.06 to 0.01	.14
Proportion negative	0.03 (0.04)	0.03 (0.04)	−0.004 to 0.01	.49
Word count	122.98 (48.90)	132.32 (64.37)	−0.46 to 19.15	.06

Abbreviation: NA, not applicable.

Similar patterns emerged from the patient messages subset. Physicians reporting burnout received a greater volume of patient messages on average (1230.0 vs 1142.9 messages), but this difference was not significant (difference, 87.1 messages; 95% CI, 363.8-189.6 messages; *P* = .54) (Table 3). There were again no significant differences in other message characteristics, including sentiment-related characteristics. Patient messages tended to be majority positive in both groups (69% among physicians with burnout and 67% among physicians without burnout). Approximately 3% of patient messages were negative in both groups.

Details of the multivariable models for burnout according to inbasket message characteristics while adjusting for physician and practice characteristics are included in Table 4. These included models of all EHR inbasket messages, as well as specifically patient messages. Identifying as Hispanic or Latino was significantly associated with increased odds of burnout (all EHR inbasket messages: odds ratio (OR), 3.44; 95% CI, 1.18-10.61; *P* = .03), as was female gender (OR, 1.60; 95% CI, 1.13-2.27; *P* = .01). Having practiced for more than 15 years was associated with decreased odds of burnout (OR, 0.46; 95% CI, 0.30-0.68; *P* < .001). Models for patient messages showed similar results (Table 4).

**Table 4.** Multivariable Models of Physician Burnout According to Analyses of All Electronic Health Record Inbasket Messages and the Subset of Patient Messages in the Inbasket, Modeling Odds of Burnout (Mini-Z Burnout Score of 3 or Greater)

Message and Physician Characteristics	All EHR inbasket messages (N = 1 453 245 messages sent to 609 physicians)		Patient messages subset (n = 630 828 messages sent to 531 physicians)	
	OR (95% CI)	*P* value	OR (95% CI)	*P* value
No. of messages	1.00 (1.00-1.00)	.83	1.00 (1.00-1.00)	.51
Mean word count per message	0.99 (0.99-1.00)	.72	1.00 (0.99-1.00)	.05^a^
Mean message sentiment score	0.14 (0.00-74.73)	.52	4.39 (0.03-800.34)	.57
Proportion of sentiment messages				
Positive	3.15 (0.57-17.71)	.19	1.99 (0.37-10.90)	.42
Negative	2.50 (0.43-15.55)	.31	1.25 (0.23-7.11)	.80
Race				
White	1 [Reference]	[Reference]	1 [Reference]	NA
Asian	0.78 (0.51-1.19)	.25	0.78 (0.49-1.23)	.28
Black	0.48 (0.09-2.11)	.34	0.62 (0.07-5.42)	.64
Prefer not to answer or other^b^	0.95 (0.44-2.06)	.89	0.97 (0.42-2.27)	.95
Ethnicity				
Not Hispanic or Latino	1 [Reference]	[Reference]	1 [Reference]	NA
Hispanic or Latino	3.44 (1.18-10.61)	.03^a^	3.50 (1.09-12.00)	.04^a^
Prefer not to answer or missing	1.58 (0.69-3.62)	.28	1.21 (0.50-2.98)	.67
Work setting				
Inpatient	1 [Reference]	[Reference]	1 [Reference]	NA
Outpatient	0.78 (0.52-1.16)	.21	0.74 (0.48-1.14)	.17
Clinical full-time equivalent				
≥50%	1 [Reference]	[Reference]	1 [Reference]	NA
<50%	1.08 (0.70-1.67)	.71	1.15 (0.72-1.84)	.55
Years in medical practice				
≤15	1 [Reference]	[Reference]	1 [Reference]	NA
>15	0.46 (0.30-0.68)	<.001^a^	0.45 (0.29-0.68)	<.001^a^
Gender				
Male	1 [Reference]	[Reference]	1 [Reference]	NA
Female	1.60 (1.13-2.27)	.01^a^	1.68 (1.16-2.46)	.01^a^
Other or prefer not to answer	1.49 (0.42-5.59)	.54	1.74 (0.49-6.69)	.40
Specialty				
Nonprimary care	1 [Reference]	[Reference]	1 [Reference]	NA
Primary care	1.45 (0.89-2.37)	.14	1.33 (0.78-2.29)	.30

Abbreviations: EHR, electronic health record; OR, odds ratio.

^a^ Denotes statistical significance at *P* < .05.

^b^ There was no secondary categorization of the “Other or prefer not to answer” category by investigators; this was self-reported by participants if they identified with a group not listed in the prespecified options.

## Discussion

The association between inbasket message sentiment and physician burnout has not been well-studied previously. In this cross-sectional study, we extracted approximately 1.5 million inbasket messages for 609 physicians from multiple specialties to better understand this potential association.

First, approximately one-half of physicians reported burnout. Physician burnout is associated with a higher rate of self-reported medical errors, depression and suicidal ideation, and departure from clinical practice.^37,38,39,40,41,42^ Physician occupational distress has also been exacerbated by the COVID-19 pandemic.^8,9,10,43,44,45,46^ Given the drawn-out nature of the pandemic, understanding factors associated with burnout and potential mitigation approaches is critical, especially given how prevalent burnout is.

Understanding interactions between EHR systems and health IT more broadly with physician burnout is a growing area of investigation. Some suggested reasons for the impact of health IT on burnout include increased time, increasing complexity and cognitive burden, poor usability, and decreased interpersonal contact with both patients and colleagues.^47,48,49,50,51,52,53,54^ In multiple studies, higher inbasket message volumes have been associated with physician burnout.^17,55,56^ Supporting those findings, we also found that physicians reporting burnout had on average higher message volumes, although in our study these differences did not reach significance.

In this study, we expanded upon the literature by using an innovative approach of using NLP to better understand inbasket message sentiment. NLP has been used in many biomedical applications, including named entity recognition, electronic phenotyping, and language generation (eg, digital health “chatbots”).^57,58,59,60,61^ Prior NLP studies have analyzed unstructured/free-text clinical notes, pathology reports, or radiology reports. According to a search of PubMed and Google Scholar databases in spring 2022, to our knowledge, there are no prior studies describing applications of NLP to EHR inbasket messages. Therefore, this analysis provides an initial understanding of inbasket message sentiment as it is associated with physician burnout, filling a critical gap.

We did not find significant associations between burnout and message sentiment. However, descriptive analyses of negative messages—the identification of which was facilitated by the NLP algorithms—still yielded several interesting findings. Analyses of high-frequency words included many expletives, demonstrating the animosity of some messages arriving at physicians’ inbaskets. Some of these messages expressed negativity specifically directed toward physicians, while others expressed frustrations at the challenges of navigating complex health care systems or laments regarding clinical conditions (Table 2). These messages could still be stressful for physicians, particularly if the patients’ frustration is related to factors beyond the individual physician’s control. However, these messages highlight the need for health systems to examine root causes of patient frustrations and improve patient engagement in their care.

There were also several high-frequency words reflecting violence or hatred. This is concerning, especially given documentation of patient-inflicted violence against physicians.^62,63,64,65^ Health systems should be proactive in ensuring that the inbasket does not become a venue for physician abuse and cyberbullying. Posting reminders in EHR patient portals to use kind language when sending messages, applying filters for expletives or threatening words, and creating frameworks for identifying patients who frequently send negative messages are potential strategies for mitigating this risk. There may also be opportunities for educating physicians how to handle electronic communications, as training on electronic communication is fairly lacking.

Finally, our multivariable models identified subgroups of physicians who may be particularly vulnerable to burnout, even when accounting for variations in message volume and sentiment. These were physicians with 15 or fewer years of medical practice, female physicians, and Hispanic/Latino physicians. Early career/younger and female physicians have been found to be at greater risk of burnout in several prior studies.^66,67,68,69^ Our finding of Hispanic/Latino physicians being associated with higher odds of burnout contrasts with a prior study^38^ that reported lower likelihood of burnout among minoritized racial and ethnic groups. However, that study analyzed data from 2017 to 2018, whereas we surveyed physicians after the onset of the COVID-19 pandemic in 2020. A more recent analysis^65^ found that mistreatment and discriminatory behaviors by patients, families, and visitors were more common among female physicians and those from racial and ethnic minoritized groups, and associated with higher burnout rates. Given widespread recognition that women and minoritized populations have been disproportionately affected by the pandemic, more research is warranted to better understand potential disparities in physician burnout.

### Limitations

This study had several limitations. First, the burnout survey was conducted at a single center, potentially limiting generalizability. However, examination of Epic Signal, a platform that provides off-the-shelf metrics regarding EHR use, demonstrates that the time in inbasket and volume of inbasket messages received at our institution is in the middle 50% of all institutions using Epic and not an outlier. Second, the burnout outcome was based on a single survey administered in spring/summer 2020, so longitudinal analyses were not possible. Additionally, the mini-Z single-item burnout measure correlates with the emotional exhaustion domain of burnout but provides less insight into the depersonalization or personal accomplishment domains. The survey was conducted during the early stages of the COVID-19 pandemic, which was associated with major changes to health care delivery. Burnout may have been underestimated as a result of survey timing, as prior studies reported low burnout rates in the first 6 to 9 months of the pandemic (38.2%),^70^ and yet a year later burnout rates were the highest ever recorded (62.8%).^10^ Follow-up studies will be needed to evaluate how the potential association between messaging characteristics and physician burnout will evolve over time. Another potential limitation was that the sentiment analysis model we used was based on a general layperson lexicon rather than a medical-clinical lexicon. We chose this lexicon due to the general nature of inbasket communications (particularly for messages from patients), but clinical terms may not have been adequately represented. Additionally, this was an observational study which is inherently limited in its ability to establish causality.

## Conclusions

We extracted nearly 1.5 million EHR inbasket messages received by 609 physicians at an academic medical center across multiple specialties to understand associations between message characteristics and physician burnout. Although we did not find significant associations between message sentiment and physician burnout, NLP facilitated automated identification of negative messages. Although infrequent, these negative messages demonstrated a striking range of hostility toward physicians and health systems in general. Using NLP for analyzing EHR inbasket messages comprises a novel approach for furthering our understanding of physician burnout and for developing strategies to reduce risk of burnout, an important consideration as federal regulation and shifting models of health care delivery during the COVID-19 pandemic have increased the use of EHR inbasket messaging. This approach can also be used in a clinical/operational context to drive quality improvement initiatives, as NLP analyses of inbasket messages at scale could potentially help institutions to identify systems issues associated with patient negativity, which, in turn, can help inform downstream interventions to improve patient experience and outcomes.

## Notes

Supplement 1. **eFigure. **Distribution of Electronic Health Record Inbasket Message Sentiment Scores **eTable. **Examples of Electronic Health Record Inbasket Messages With Positive and Negative Sentiment Scores **eAppendix. **Survey

Supplement 2. Data Sharing Statement
