# Supplementary material for: Association of Electronic Health Record Inbasket Message Characteristics With Physician Burnout
Source: JAMA Netw Open. 2022 Nov 30;5(11):e2244363. doi: 10.1001/jamanetworkopen.2022.44363 (PMC9713605; doi:10.1001/jamanetworkopen.2022.44363)
Supplement: Supplement 1. — eFigure. Distribution of Electronic Health Record Inbasket Message Sentiment Scores eTable. Examples of Electronic Health Record Inbasket Messages With Positive and Negative Sentiment Scores eAppendix. Survey [file jamanetwopen-e2244363-s001.pdf]

Supplemental Online Content

Baxter SL, Saseendrakumar BR, Cheung M, et al. Association of electronic health record inbasket message characteristics with physician burnout. *JAMA Netw Open*. 2022;5(11):e2244363. doi:10.1001/jamanetworkopen.2022.44363

eFigure. Distribution of Electronic Health Record Inbasket Message Sentiment Scores
eTable. Examples of Electronic Health Record Inbasket Messages With Positive and Negative Sentiment Scores
eAppendix. Survey

This supplemental material has been provided by the authors to give readers additional information about their work.

© 2022 Baxter SL et al. *JAMA Network Open.*

eFigure. Distribution of Electronic Health Record Inbasket Message Sentiment Scores

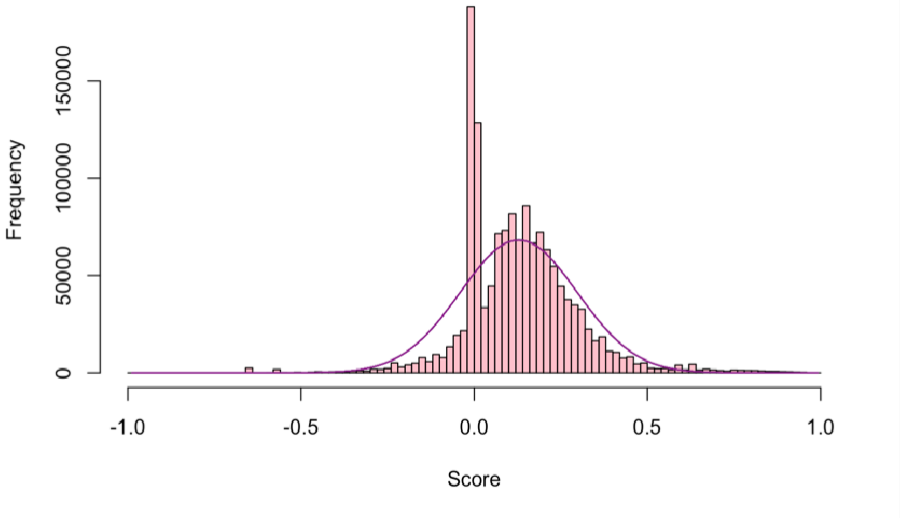

© 2022 Baxter SL et al. *JAMA Network Open.*

eTable. Examples of Electronic Health Record Inbasket Messages With Positive and Negative Sentiment Scores

Dates and names have been removed and replaced with asterisks.

Examples of Positive Sentiment Messages	Examples of Negative Sentiment Messages
Good Morning, I hope everything is well with you and your family. It took me a while to send a letter. I have to adjust to our new Normal. Everything is great with us. I would like you to know that my whole family is grateful for taking care of US.<br>The last 9 months was A journey but because of your guidance and care it became an ease.<br>Peace of Mind is something that you can't buy and you gave that to us, because you are my MD. Words are not enough. Checking on us after the delivery is an icing on top. Please always remember on a hard day, that you are helping a lot of people doing what you do BEST. You are a BLESSING to all of US and OBGYN and GYNO at UCSD. Have a Great week ahead. My family loves you. Thanks<br>Again for helping me with 2 Wonderful Gifts.	I am in real trouble. I am in so much pain, I am in tears. I can barely walk. Im passing out all over my house from severe exhaustion. My feet and legs feel like they are going to split open. My thighs, lower back and kidneys are in so much pain. My upper back is just aching badly. My whole body is in such incredible pain. My Cluster Headaches are killing me. Just had the worst attack I can remember. I feel like whatever is happening to me is going to kill me. My arms are swollen and my left arm/elbow feels like it is fractured. Dr. *** office said I need to do a rest and exercise test. Doc I can't go anywhere let alone any appointments. I can barely walk. I have never been in this much pain in my life. I need help badly. I need to be back on hospice now
Thank you. thank you for your care. thank you for your incredible service to me and the thousands and millions of lives you touch every day. I am thinking of you today because I am well. I think I'm well because of the amazing care I receive from you. I'm 'of an age' and have asthma and high blood pressure. All things are well in my life. I am hope, self-isolating for the duration. I have the pleasure of working at UC San Diego main campus, and am lucky to work remotely. I am very blessed with a healthy and self-isolating family - 5 different locations. So I want to make sure I thank you for all you are. I am blessed with you in my life.	I am so glad you are going to continue to see patients! While you were temporarily out (on **), I came into the office and saw a nurse about my consisent discomfort, bloating of my lower abdomen since the colonoscopy on **. Both cancer doctors assure me that it has nothing to do with my<br>cancer. She guessed IBS (see notes). Since then, I had a painful night on ** - extreme pain which resulted in nausea, vomitting, and bowel movements. The left side<br>stabbing pain would occur about every 15 minutes. After 6-7 times, I was dry heaving and my bowel movements had become liquid/watery. Then after about 2 1/2 hours it stopped. When I woke up after about 3 hours of sleep, I had a lot of blood in the small amount of bowel movement left at my anal opening (maybe my cancer tumor?). Anyway, one of my cancer doctors asked me if I had ever been diagnosed with DIVERTICULOSIS. I was in the colonoscopy of **. The<br>recent one concentrated more on my cancer
I just got off the phone with Mr *** and I had to pass on his comments. He said that he was treated better than the president! His whole experience from his pretty and professional nurse to everyone he met that day treated him like he was the most important person in the world. He said your department was the best he has experienced and his driver was so impressed that he said he was going to change<br>his care to UCSD. I appreciate your professionalism and your awesome collegiality. Thank you for taking such great care of our patient's and making them feel so well cared for!	Did you screen me for pelvic inflammatory disease? That can also explain some of the symptoms I have been exhibiting for quite some time with pelvic pain. Like I have said, my pelvic pain got worse after my endometrial ablation and changing my diet almost three years ago. Today when I stretched my hip in physical therapy I was in so much pain I wanted to cry and puke at the same time. The pain radiated from my back, left hip, and down my leg into my knee. The whole left side of my pelvis felt like it was on fire. I still hurt when I sit, but this issue has been on and off. I figured I would let you know about the pain today at least. I only noticed my hip pain get worse since I had increased my exercise over the last six months, but the left side pain had been increasing since the ablation. Bowel changes happened more after I changed my diet<br>and dropped a lot of weight. All the problelms I had when you treated me for vaginosis are still around.

© 2022 Baxter SL et al. *JAMA Network Open.*

AMA Survey Final

Start of Block: Intro screen

Q1 Thank you for your interest in participating in our study! Please take a moment to confirm the following: are you an attending physician at UC San Diego?

○ Yes, I am an attending physician at UCSD. (1)

○ No, I am not an attending physician at UCSD. (2)

End of Block: Intro screen

Start of Block: Consent Block

Q2 Welcome to our study of physician wellness and coping with COVID-19. Please click on the link below to access the informed consent in a new screen. You can close the consent form page to return to the survey.

Informed Consent

Q3
By clicking “You agree” below you are indicating that you are at least 18 years old, have read this consent form, and agree to participate in this research study.

○ You agree (3)

○ You do not agree (4)

End of Block: Consent Block

Start of Block: Full survey question block

Q4 Thank you for agreeing to participate. Please enter your UCSD email address below.

○ username@health.ucsd.edu (4) ____

Page Break

© 2022 Baxter SL et al. *JAMA Network Open.*

Q5 Coping with COVID-19 Survey

The following questions assess the possible impact of COVID-19 on healthcare workers.

Q6 Your question here

	Minimal (1)	Modest (2)	High (3)	Very High (4)
1) The stress I experienced today is (2)	○	○	○	○

Q7 Your question here

	Not at all (1)	Somewhat (2)	Moderately (3)	To a Great Extent (4)
2) I worry about exposing myself and my family to COVID-19 (1)	○	○	○	○

Q8 3) Due to the impact of COVID-19, I am experiencing the following:

	Not at all (1)	Somewhat (2)	Moderately (3)	To a Great Extent (4)
Anxiety or depression (1)	○	○	○	○
Work overload (2)	○	○	○	○
Concerns about childcare (3)	○	○	○	○

Q9 If you are feeling severe distress, please contact the UC San Diego Health Healer Education Assessment and Referral Program (HEAR) at hear.ucsd.edu or 858-657-6795, local mental resource, or the national suicide prevention helpline at 1-800-273-8255.

© 2022 Baxter SL et al. *JAMA Network Open.*

Q10
4) How would the following improve your ability to sustain through the COVID-19 crisis?

	Not at all (1)	Somewhat (2)	Moderately (3)	To a Great Extent (4)
Staff or colleague support for inbox (1)	○	○	○	○
Healthy food available at all hours (2)	○	○	○	○
Personal access to mental health care (3)	○	○	○	○

Q11 Your question here

	Not at all (1)	Somewhat (2)	Moderately (3)	To a Great Extent (4)
5) Being part of the COVID-19 response has increased my sense of meaning and purpose. (1)	○	○	○	○

Q12 Your question here

	Not at all (1)	Somewhat (2)	Moderately (3)	To a Great Extent (4)
6) I feel valued by my organization. (1)	○	○	○	○

Q13 7) What else would you like to tell us about your experience during the COVID-19 crisis?

(Note: anonymous and aggregate responses may be shared with UC San Diego Health's leadership team to inform decision-making.)

© 2022 Baxter SL et al. *JAMA Network Open.*

____

____

____

____

____

Page Break

© 2022 Baxter SL et al. *JAMA Network Open.*

Q14 Physician Wellness Survey

Please think about your work in general, and answer the following questions.

Q15 Your question here

	Agree strongly (1)	Agree (2)	Neither agree nor disagree (3)	Disagree (4)	Strongly disagree (5)
8) Overall, I am satisfied with my current job. (1)	○	○	○	○	○

Q16 9) Using your own definition of “burnout," please choose one of the numbers below*

○ 5 = I enjoy my work. I have no symptoms of burnout. (1)

○ 4 (2)

○ 3 = I am beginning to burn out and have one or more symptoms of burnout, e.g. emotional exhaustion. (3)

○ 2 (4)

○ 1 = I feel completely burned out. I am at the point where I may need to seek help. (5)

Q17 *If you select 1 or 2, please consider seeking assistance – call your healthcare provider or UCSD's HEAR program.

© 2022 Baxter SL et al. *JAMA Network Open.*

Q18 Your question here

	Agree strongly (1)	Agree (2)	Neither agree nor disagree (3)	Disagree (4)	Strongly disagree (5)
10) My professional values are well aligned with those of my clinical leaders. (1)	○	○	○	○	○

Q19 Your question here

	Poor (1)	Marginal (2)	Satisfactory (3)	Good (4)	Optimal (5)
11) The degree to which my care team works efficiently together is (1)	○	○	○	○	○

Q20 Your question here

	Poor (1)	Marginal (2)	Satisfactory (3)	Good (4)	Optimal (5)
12) My control over my workload is (1)	○	○	○	○	○

© 2022 Baxter SL et al. *JAMA Network Open.*

Q21 Your question here

	Agree strongly (1)	Agree (2)	Neither agree nor disagree (3)	Disagree (4)	Strongly disagree (5)
13) I feel a great deal of stress because of my job. (1)	○	○	○	○	○

Q22 Your question here

	Excessive (1)	Moderately high (2)	Satisfactory (3)	Modest (4)	Minimal/none (5)
14) The amount of time I spend on documentation is (1)	○	○	○	○	○

Q23 Your question here

	Excessive (1)	Moderately high (2)	Satisfactory (3)	Modest (4)	Minimal/none (5)
15) The amount of time I spend on the electronic medical record (EMR) *at home* is (1)	○	○	○	○	○

© 2022 Baxter SL et al. *JAMA Network Open.*

Q24 Your question here

	Excessive (1)	Moderately high (2)	Satisfactory (3)	Modest (4)	Minimal/none (5)
16) The amount of frustration I experience with the EMR during my day (1)	○	○	○	○	○

Q25 17) Which number best describes the atmosphere in your primary work area?

○ 5 = Calm (1)

○ 4 (2)

○ 3 = Busy, but reasonable (3)

○ 2 (4)

○ 1 = Hectic, chaotic (5)

Q26 Your question here

	Strongly disagree (1)	Disagree (2)	Neither agree nor disagree (3)	Agree (4)	Agree strongly (5)
18) I am likely to reduce my FTE in the next 12 months. (1)	○	○	○	○	○

© 2022 Baxter SL et al. *JAMA Network Open.*

Q27 19) How true is the following statement about conditions in your principal practice site?

	Not at all true (1)	Somewhat true (2)	Moderately true (3)	Very true (4)	Completely true (5)
Physicians are highly valued. (1)	○	○	○	○	○

Q28 20) In the past 2 weeks,

How many hours of sleep did you get each night? (1)	▼ <5 hrs (1) ... >8 hrs (5)
How many days/week did you exercise for 30 cumulative minutes or more? (2)	▼ <5 hrs (1) ... >8 hrs (5)
How many days/week did you practice mindfulness (e.g. meditation, breathing exercises, gratitude practice)? (3)	▼ <5 hrs (1) ... >8 hrs (5)

Page Break

© 2022 Baxter SL et al. *JAMA Network Open.*

Q29 Demographic Information

Please respond to the following demographic questions.

Q30 21) Please specify your gender.

- ○ Male (1)
- ○ Female (2)
- ○ Non-binary/Third gender (3)
- ○ Prefer not to answer (4)

Q31 22) What is your race?

- ○ Black or African American (1)
- ○ Asian (2)
- ○ Native American (3)
- ○ White (4)
- ○ Native Hawaiian or other Pacific Islander (5)
- ○ Prefer not to answer (6)

Q32 23) What is your ethnicity?

- ○ Latino/Hispanic (1)
- ○ Not Latino/Hispanic (2)
- ○ Prefer not to answer (3)

© 2022 Baxter SL et al. *JAMA Network Open.*

Q33 24) Overall, do you consider yourself to work

- ◯ Full-time (1)
- ◯ Part-time (2)

Q57 25) Please indicate your percentages of clinical and administrative FTE below.

	0 10 20 30 40 50 60 70 80 90 100
Clinical FTE (1)	
Administrative FTE (2)	

Q35 26) In what type of setting do you spend the majority of your clinical time?

▼ Inpatient (1) ... Outpatient (2)

Q36 27) How many years after training have you been in practice?

- ◯ 1-5 years (1)
- ◯ 6-10 years (2)
- ◯ 11-15 years (3)
- ◯ 16-20 years (4)
- ◯ More than 20 years (5)

Q37 28) Please indicate the Department of your primary appointment at UC San Diego School of Medicine.

▼ Anesthesiology (1) ... Urology (18)

© 2022 Baxter SL et al. *JAMA Network Open.*

Display This Question:

If 28) Please indicate the Department of your primary appointment at UC San Diego School of Medicine. = Medicine

Q38 29) Please select your Division in the Department of Medicine.

▼ Blood and Marrow Transplantation (1) ... Rheumatology, Allergy, & Immunology (15)

End of Block: Full survey question block

© 2022 Baxter SL et al. *JAMA Network Open.*
