# Supplementary material for: Association of Electronic Health Record Inbasket Message Characteristics With Physician Burnout
Source: JAMA Netw Open. 2022 Nov 30;5(11):e2244363. doi: 10.1001/jamanetworkopen.2022.44363 (PMC9713605; doi:10.1001/jamanetworkopen.2022.44363)
Supplement: Supplement 2. — Data Sharing Statement [file jamanetwopen-e2244363-s002.pdf]

Data Sharing Statement

Baxter. Association of Electronic Health Record Inbasket Message Characteristics With Physician Burnout. *JAMA Netw Open*. Published November 30, 2022. doi:10.1001/jamanetworkopen.2022.44363

Data
Data available: No

Additional Information
Explanation for why data not available: Combinations of the data may be able to identify physicians at our institution with burnout based on demographic characteristics, specialty, etc. Due to the sensitive nature of burnout and medical practice, we did not want to share this data for concern of re-identification.
